# Editorial: Shape and size dependent nanostructures for environmental applications

**DOI:** 10.3389/fchem.2024.1362033

**Published:** 2024-01-22

**Authors:** Basudev Baral, Ali Altaee, Konstantinos Simeonidis, Akshaya K. Samal

**Affiliations:** ^1^ Centre for Nano and Material Sciences, Jain University, Jain Global Campus, Ramanagara, Bangalore, Karnataka, India; ^2^ Centre for Green Technology, School of Civil and Environmental Engineering, The University of Technology Sydney, Sydney, NSW, Australia; ^3^ Department of Chemical Engineering, School of Physics, Aristotle University of Thessaloniki, Thessaloniki, Greece

**Keywords:** nanostructures, dimensions, morphology, green energy, environmental applications and catalysis

**FIGURE 1:**
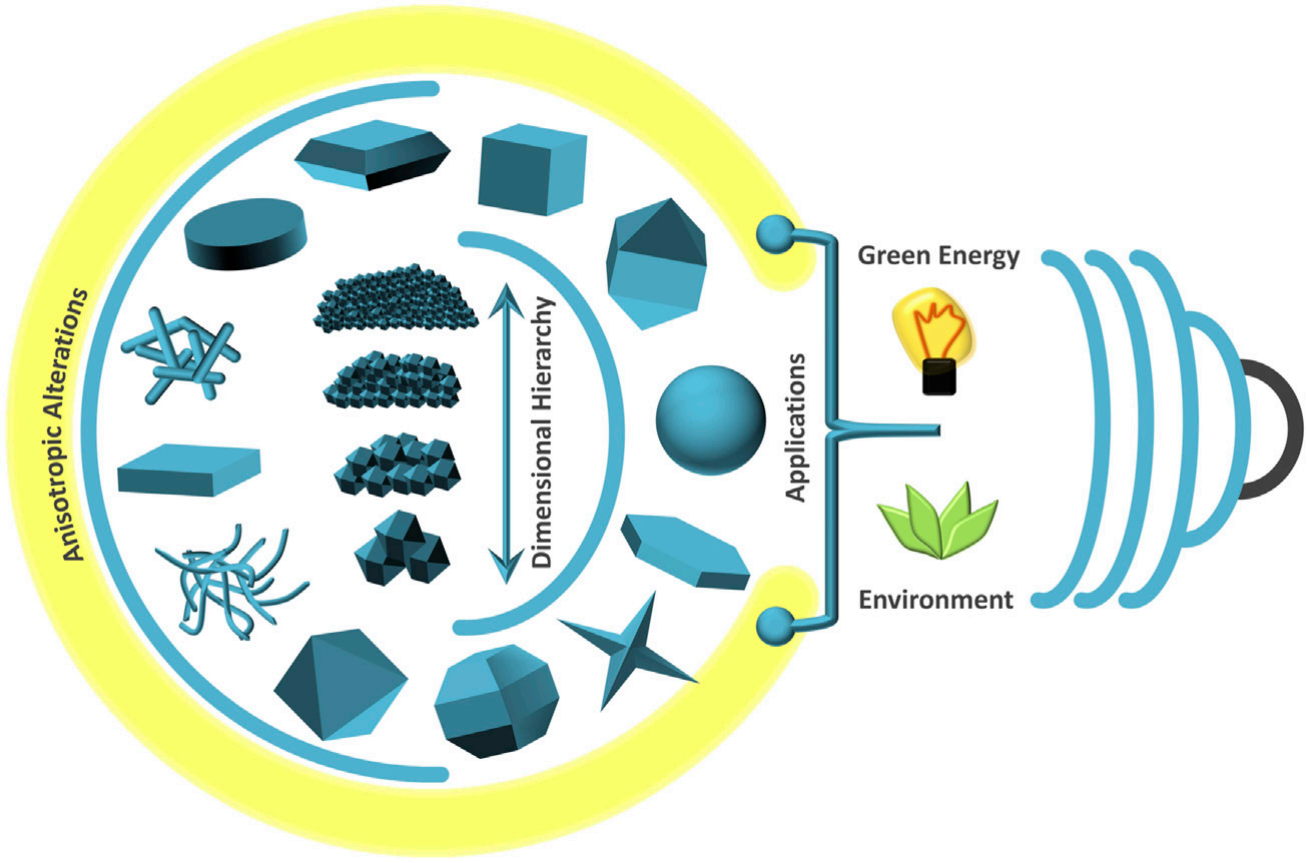
Nanostructured materials with anisotropic alterations and dimensional hierarchy for green-sustainable energy-environmental applications.

The cooperative chase to comprehend the dynamical role of functional nanostructures over the immense backdrop of environmental remediation has taken the cornerstone of the current topic “*Shape and size dependent nanostructures for environmental applications*”. Surmounting the conventional precincts, the issue has aimed to platform hot research, perspectives, refined reviews and mini-reviews with legitimate impact. In this context, explanatory staging of nucleation and growth dynamics of nanostructures, exploration of relative reaction selectivity, synchronous monitoring of nano-micro factors distressing the reactivity and elaborative demarcation of effectual association of anisotropic and dimensional aspects over ultimate competence, are taken as major headlines for the contents of the issue. Moreover, exploring innovative synthesis tactics, nano-micro level scientific insights, and compressive environmental applications were selectively chosen as the moral integrity of the topic. The promising lead contributing articles of the issue illustrate synergy among anisotropic and dimensional factors, ecological relevance, efficiency and sustainability of nanostructured materials in a pervasive way. The current editorial propagates through the contributions of unique research upshots further expanding the acquaintance on anisotropic and dimension-controlled materials for energy-environmental confronts paving the way for future novel research and discoveries (Figure 1).

The advent of functional nanostructured materials has gained astounding anticipation as a green-sustainable elucidation of Energy-Environmental deluge repercussioned via man-made blunders. Surveying recent breakthroughs in nanostructure research unveils a multitude of applications. The employment of nanostructured materials as chemoelectric sensors, as detectors and as nanoelectronics has brought a tremendous insurrection in global environmental monitoring, advanced hazard detection and emission control processes. Furthermore, the remarkable role of nanoscale materials in global bio-environmental air-water-soil contamination treatment via adsorption, filtration, conversion and degradation of poisonous gases, radioactive wastes, organic-inorganic chemicals, elemental hazardous materials and micro-pathogens is invincible (Khin et al., 2012; Mauter and Elimelech, 2008). Furthermore, nanomaterial-based photovoltaic, photocatalytic, photoelectrocatalytic and photothermocatalytic material architecture for sustainable power generation has restructured the green energy segment (Swain et al., 2023). Advancing further, nanostructured materials-based supercapacitors and efficient battery technology have taken enormous attention in green energy storage research (Bhol et al., 2023; Mousavi et al., 2023). In this context, nanostructures have revolutionized environmental applications by offering a spectrum of opportunities to tackle pressing issues.

Such endeavour has underpinned the architecture of copious dynamic nano-micro structured materials with manoeuvred physio-physical-electrochemical attributes. Out of the assorted approaches, the catalytic application of nanostructured materials for energy-environmental remediation has evolved as a dexterous technology. The mainstay criterion of such splendid research is to engineer a functional material with elevated quantum yield, proficient conversion efficiency, and relegated disbursement. Conversely, the conventional physical-biological procedures are associated with inescapable boundaries. In this context, the meticulous fabrication of nanostructured material with controlled size and morphology is established to achieve superior catalytic efficiency. The shape and size-dependent properties of nanostructures offer a myriad of potential for tackling environmental challenges.

The morphology and dimension of nano-structured materials strongly hamper the dynamics of surface-coupled catalysis. The pivotal role of the surface/volume ratio of nanostructured particles with copious sizes towards manipulated catalysis behaviour is well evidenced (Zhou et al., 2009). Drastic alteration in size and morphology impacts the overall physicochemical and electrochemical properties. Anisotropic growth of crystals demonstrates the exposure of diverse active surfaces (Gupta et al., 2023; Baral et al., 2021), alteration in bond energy value, the conception of electron localization/delocalization centres (Madhu et al., 2023), exciton injection via lattice mismatch (Kundu et al., 2023) and alteration in active surface area (Konar et al., 2023) like parameters. Morphology disparity strictly facilitates distortion in atomic arrangement and steric constraints, thus impacting the overall chemo-absorption course (Wu et al., 2021). Desirable surface coordination of catalytically active atoms or domains substantially impacts the ultimate catalytic activity, selectivity and stability (Li and Shen, 2014).

On the other hand, selective exposure of high and low-indexed polar functional facets actively alters the facet-oriented catalytic interaction, coordinative unsaturated sites, surface oxygen mobility, surface oxygen vacancy, and acidic-basic sites responsible for diverse catalytic activity and selectivity (Baral and Parida, 2020). Nevertheless, the diverse electron mobility over selective facets alters the electro-oxidation-reduction process in a superior manner compared to traditional counterparts (Xie and Shen, 2009; Khobragade et al., 2021). Moreover, selective surface exposure with a high concentration of surface oxygen vacancy was reported to drastically convert the lattice oxygen to active oxygen and thus alter the ultimate activity (Zheng et al., 2019).

Accounting for the effectual association of catalyst dimension towards catalysis, it was precisely documented that diminutive particle size supports rapid charge transfer dynamics via minimal charge diffusion path and deteriorated resistance (Dong et al., 2018). Nevertheless, size-controlled nano-structured particles self-assembled to create the foundation to facilitate crystalline array formation and thus impact the reactivity. Drastic modification in particle size altering population of defect sites responsible for elevated catalysis over Au nanoparticles, is well authenticated by (Liang et al., 2022). The dimension of metal nanoparticles contributes to the electronic properties and plays a vital role in reactant adsorption, activation and desorption phenomena (Li et al., 2011). Disentangling physicochemical and electronic attributes of nano-structured catalysts exposes new concourse for catalyst fabrication (Wang et al., 2019).

With colossal contentment in this forum issue, we are emphasising and collectively conveying some of the hot innovations and critical review studies on the area of morphology and controlled dimension-dependent nanostructure-based catalysis. These contributions exemplify how introducing anisotropic features and controlling the catalyst dimensions can neatly expand the physicochemical attributions of nanocatalytic materials.

Plasmonic absorbance and emission are consequences of the size and dimension of the noble metals. In this regard, diverse plasmonic noble metals have been meticulously architected (Larsen et al., 2016; Bhavya et al., 2020). Advancements in developing bimetallic plasmonic alloys have also been achieved to surmount associated metal-oxidations like shortcomings (Vega et al., 2021). Combining size-controlled noble plasmonic materials with secondary shape-selective nanostructures can be a constructive avenue in catalysis as interaction and stabilization of metallic ions over specific surfaces of nanomaterials plays a vital role towards ultimate catalytic activity (Lykaki et al., 2018). In this context, the fabrication of size-controlled noble metals and successive hybridization over core catalytic material can efficiently elevate the overall activity to a demonstrable level (Gao et al., 2014; Yin et al., 2018; Herring and Montemore, 2023). Carbonaceous materials like fullerene, graphene, and carbon nanotubes with higher active surface area also grabbed the attention as efficient substrate cum support material for dimension-controlled plasmonic nano metals (Lam and Luong, 2014; Zhang et al., 2014; Baral et al., 2021). A proper assortment of confounding active surface area, elevated conductivity, and graphitic structure invent carbonaceous materials as magnificent support material for the core catalysts (Sun et al., 2013).

The desire to synthesize an improved size controlled noble bimetallic alloy and further hybrid formation with carbonaceous material and morphology triggered metal oxides pinned the foundation for the development of a noble bimetallic alloy: carbon nanotube: anisotropic metal oxide based ternary hybrid catalyst.

In a viewpoint article, Gyves et al. validated the consequential effect of modification of Au-Cu bi-metallic alloy over multiwalled carbon nanotubes (MWCNTs), and CeO_2_-nanobar. Nevertheless, comparative studies by intriguing CeO_2_–nanorod in place of CeO_2_-nanobar was also carried out towards electrocatalytic glycerol (sustainable material for fuel cell) oxidation analysis in alkaline media (Simões et al., 2010). A clear illustration of the effect of morphology and dimension on catalysis was well documented.

The importance of dimension-controlled nano metallic alloys is further articulated and exemplified by Mi et al. Despite scores of improvement, numerous uncertainties remain at the forefront, and organised data is limited. In accordant with this, a quality review work was reported emphasizing and summarizing the vital points like current development on Au–Cu nanostructures, controlled-meticulous fabrication strategies, emerging applications and future prospective.

In the vast dimension of catalysis, polymers have evolved as expectant active catalytic and support materials. Undoubtedly, controllable textural cum morphological parameters attributing to changeable electronic features make polymers inimitable candidates. Auxiliary porosity creation expands the dynamic surface area of polymers and hence elevates the activity (Kaur et al., 2011; Sun et al., 2015). Finely, tuning catalyst-polymer structure−property relationships can alter the dynamics of the catalytic process (Madhavan et al., 2008; Liu et al., 2012). In this context, introducing dimensional and morphological hierarchy over polymeric materials can sternly amend the absolute activity. Accounting this, the augmented efficiency of hollow-structured Prussian white (PW) polymer with an elevated surface area compared to cubic crystals is well attested by (Bu et al., 2014).

In a featured study, Bosacka et al. neatly explored the fabrication and characterization of an S,S′-thiodi-4,1-phenylene bis(thio-methacrylate)-co-divinylbenzene (DMSPS-co-DVB) nanopolymeric catalyst materials with varying DMSPS and DVB proportions. Moreover, the effectual association of synthetic strategy, morphology/dimension, and aniline absorption kinetics are well ascertained.

Moreover, the drastic accent of exhausted CO_2_ concentration in the open atmosphere is a terrible concern. Despite the fact that diverse techniques like photocatalytic, electrocatalytic and thermocatalytic CO_2_ fixation have evolved, catalytic CO_2_ remediation is still a sizzling Research Topic and an imperative requisite. Nevertheless, renewable CO_2_ offers a promising source of low-carbon-footprint fuels and chemicals (Xie et al., 2022). Accounting for the preposterous conversion efficiency associated with the CO_2_ condensation technique, catalytic remediation appears to be a more utilizable and viable technology as it proceeds via a catalytic reduction to multi-carbon (C_2+_) products like formic acid, methanol, oxidative coupled methane, oxidative dehydrogenated ethylene, and carboxylic acids, etc (Yamazaki et al., 2022; Burkart et al., 2019; Von Der Assen et al., 2013).

Considering the mere importance of catalytic CO_2_ utilization, Kandathil and Manoj articulated a review by briefing the diverse chemical approaches for the developments of anisotropic nanomaterials. Furthermore, the group has evidently illustrated the strategic applications of the anisotropic nanomaterials in CO_2_ catalytic conversion, the associated recompense, drawbacks, and future challenges.

Along with individual scientific achievement, the research topics accorded over this forum concurrently expose capsulated knowledge and assemble a new-fangled trail for future technological advancement in the associated field. Innovative material architecture, scientific interrelationship and systematic articulation of pre-established research work demonstrated a more exhaustive perception of the topic with cavernous scientific cognisance. The scope of the journal, along with the heterogeneity of embellished articles, suggests the encyclopedic spectrum of this issue.

Last and foremost, the authors and reviewers are deeply appreciated for the fine articulation and refinement of the research upshots associated with the topic “Shape and Size Dependent Nanostructures for Environmental Applications”. We anticipate that the topic will attract scientific literates and investigators nourishing them with novel research insights and will provide them with some cerebral stimulation.
